# A new home for *Human Genomics*

**DOI:** 10.1186/1479-7364-6-1

**Published:** 2012-07-05

**Authors:** Vasilis Vasiliou, Daniel W Nebert

**Affiliations:** 1 Department of Pharmaceutical Sciences, University of Colorado Anzchutz Medical Campus, Aurora, CO, 80045; 2 Department of Environmental Health, University of Cincinnati Medical Center, Cincinnati, OH, 45267

## 

We live in exciting times. Mind-blowing advances in high-throughput technology are occurring at an ever-increasing rate. Next-generation sequencing methods (both whole-exome and whole-genome) are generating an explosion in the amount of new bioinformatics data.

Since mid-2010, genome-wide exome sequencing has successfully identified the mutated gene for a number of rare autosomal recessive and X-linked disorders. Whole-genome sequencing of a number of different cancers, especially combined with comparison of DNA sequence from the normal corresponding cell type in the same patient, has revealed a number of novel variants in genes participating in genetic networks that appear to be associated with the tumorigenesis process. A recent achievement is the whole-genome sequencing from patients with advanced breast cancer, thereby leading to the identification of distinct cancer ‘signatures’ that may predict therapeutic outcome in women on estrogen-lowering therapy, while preventing others from receiving unnecessary treatment. Advances in human genome analysis, metabolomics (using combinations of ultra-high-pressure liquid chromatography, mass spectrometry, and nuclear magnetic resonance spectroscopy), transcriptomics, epigenetics (DNA-methylation patterns, microRNA chips, histone modifications, and chromatin remodelling) and drug discovery using high-throughput screening—all will enhance our understanding and elucidation of human complex diseases and should improve diagnosis and treatment.

We wish to welcome you to Volume 6 of *Human Genomics* with exciting news. The *Human Genomics* journal is now under BioMed Central as a peer-reviewed, open-access, online journal. *Human Genomics* will continue to focus on applications of genomic and epigenomic analyses in understanding and elucidation of human complex diseases as well as other multifactorial traits. This will include diagnosis, treatment and intervention in human common diseases—as well as better understanding of Mendelian diseases, adverse drug reactions, drug efficacy and safety. Our editorial board remains virtually the same, with a few additions coming up in the near future. All the back-content issues (Volumes 1–5) of *Human Genomics* are now freely available on the journal's website.

*Human Genomics* is rather unique to other journals with its position at the interface between Big Pharma and academic research, publishing primary research and review articles relevant to both, and arising from both industry and academia. Areas that are covered, but are not limited to, include the following: pharmacogenomics, genome-wide association studies, genome-wide exome sequencing, next-generation whole-genome sequencing, functional genomics, translational genomics, expression profiling, epigenomics, proteomics, bioinformatics, animal models, statistical genetics, genetic epidemiology, human population genetics and comparative genomics.

We will also continue to have our highly successful mini-reviews on (i) *Gene Family Updates*, (ii) *Software Reviews* and (iii) *Genome Databases*.

*Gene Family Updates* are mini-reviews that examine important human gene families and, if needed, recommend the most appropriate gene family nomenclature—based upon divergent evolution.

*Software Reviews* describe and evaluate critical software recently available, e.g. analysis of large genome-related datasets (genome-wide association studies, next-generation sequencing copy-number variant patterns, DNA-methylation examples, microRNA chip assays, linkage analysis, gene-expression microarray and RNA-sequencing studies, genetic variants and genomic variability, assembly, annotation, phylogenetic analysis, gene structure and genetic architecture).

*Genome Databases* represent mini-reviews describing and/or evaluating databases, thereby providing information regarding the human genome (DNA sequences, microRNAs, large-effect vs. small-effect genes, genomic variability, comparative genomics, human diseases, pharmacogenetics, pharmacogenomics, adverse drug reactions, individualised drug therapy, personalised medicine, etc.).

Welcome to *Human Genomics*!

